# Clinical associations of *ESR2* (estrogen receptor beta) expression across thousands of primary breast tumors

**DOI:** 10.1038/s41598-022-08210-3

**Published:** 2022-03-18

**Authors:** Hina Dalal, Malin Dahlgren, Sergii Gladchuk, Christian Brueffer, Sofia K. Gruvberger-Saal, Lao H. Saal

**Affiliations:** 1 Division of Oncology, Department of Clinical Sciences Lund, Lund University Cancer Center, Lund University, Medicon Village 404-B2, 22381 Lund, Sweden; 2 Lund University Cancer Center, Medicon Village, Lund, Sweden; 3 Present Address: Section for Molecular Diagnostics, Skåne University Hospital, Lund, Sweden

**Keywords:** Breast cancer, Endocrine cancer, Tumour biomarkers, Predictive markers, Translational research

## Abstract

Estrogen receptor alpha (ERα, encoded by *ESR1*) is a well-characterized transcription factor expressed in more than 75% of breast tumors and is the key biomarker to direct endocrine therapies. On the other hand, much less is known about estrogen receptor beta (ERβ, encoded by *ESR2*) and its importance in cancer. Previous studies had some disagreement, however most reports suggested a more favorable prognosis for patients with high *ESR2* expression. To add further clarity to *ESR2* in breast cancer, we interrogated a large population-based cohort of primary breast tumors (n = 3207) from the SCAN-B study. RNA-seq shows *ESR2* is expressed at low levels overall with a slight inverse correlation to *ESR1* expression (Spearman R = −0.18, p = 2.2e−16), and highest *ESR2* expression in the basal- and normal-like PAM50 subtypes. *ESR2*-high tumors had favorable overall survival (p = 0.006), particularly in subgroups receiving endocrine therapy (p = 0.03) and in triple-negative breast cancer (p = 0.01). These results were generally robust in multivariable analyses accounting for patient age, tumor size, node status, and grade. Gene modules consistent with immune response were associated to *ESR2*-high tumors. Taken together, our results indicate that *ESR2* is generally expressed at low levels in breast cancer but associated with improved overall survival and may be related to immune response modulation.

## Introduction

Breast cancer (BC) is the most frequently diagnosed cancer in women worldwide^1^ and although the 5-year prognosis is good, it remains a public health issue on a global scale as it has overtaken lung cancer as the most commonly diagnosed cancer in the world according to recent global cancer estimates^2^.

Three quarters of all breast cancers are positive for expression of estrogen receptor alpha (ERα), encoded by the *ESR1* gene^3^, making ER signaling the most important target of clinical treatments in ERα-positive BC. The effects of estrogen are also mediated by estrogen receptor beta (ERβ) encoded by *ESR2*^4^. The mechanisms of ERα signaling in BC has been well studied over the past decades, with high expression being a potent driver of dysregulated endocrine signaling at multiple levels in BC^5–8^. While the role of ERα/*ESR1* is largely established^9–11^, the potential therapeutic role and the extent of involvement of ERβ/*ESR2* in treatment, progression and prognosis of BC remains uncertain^12–16^.

ERβ has been found to be expressed in normal breast epithelial cells as well as in various other tissues such as uterus, ovary, prostrate and brain, as well as in breast cancer cell lines^17–20^. The role of ERβ in breast cancer has been studied in various in vivo and in vitro models, suggesting its contribution in inhibiting BC tumor progression and its potential role as tumor suppressor. In cell models, ERβ has been found to enhance the response to tamoxifen^21,22^ and ERβ selective agonists reduce anti-apoptotic signaling^23^. ERβ activation increases cell autophagy^21,24^ and the generation of reactive oxygen species^22^ which may be part of the explanation for these results. Conversely, ERβ has been found to decrease the response to cytotoxic agents such as cisplatin, paclitaxel and doxorubicin^21^ and in triple-negative cell lines, enhances the antiproliferative effects of raloxifene^25,26^ and increases sensitivity to anti-androgens^27^.

Reports on the prognostic value of ERβ are conflicting. On one hand, some studies showed that high ERβ expression, irrespective of the ERα status, is a treatment response marker for BC patients receiving chemotherapy^14,28,29^ and endocrine therapy^29–32^. On the other hand, some report the opposite, where increased expression of ERβ in patients receiving endocrine therapy predicted poor prognosis and significantly reduced median tumor-free survival time^33^ as well as lower disease-free survival (DFS) in postmenopausal primary BC patients^34^. The association to poor prognosis was reported in particular for patients with triple-negative breast cancer (TNBC)^35–37^, but even in this subgroup, some studies have indicated a favorable prognosis^38,39^. Other studies reported no remarkable association between ERβ expression and patient outcome^40,41^.

Part of the reason for the conflicting results may be the lack of standardized methods for detecting ERβ in immunohistochemical (IHC) analysis and variable performance of the antibodies utilized across the many studies. Andersson et al. applied rigorous methods for validating commonly used ERβ antibodies and found that only one out of thirteen was specific for ERβ and that expression levels in human tissues were accordingly lower than previously reported^42^. Inadequate validity and poor specificity of ERβ antibodies has been an issue in much of the literature on ERβ protein expression, highlighted by the finding that neither of the well-studied purported ERβ-positive cancer cell lines, MCF-7 (breast) and LNCaP (prostate), expressed any ERβ when using validated antibodies and independent mass spectrometry-based approaches^43^. To address these issues, there have been efforts to standardize IHC protocols^19^ which can serve as a reference for future antibody-based ERβ studies.

Hence, the involvement and importance of ERβ (*ESR2*) in breast cancer remains controversial; moreover, most studies have been focused on measuring ERβ protein levels. In this study, we set out to characterize *ESR2* mRNA expression levels and investigate its association to clinicopathological features and patient outcomes. To accomplish this, we analyzed gene expression in a large, population-based cohort of 3207 primary invasive breast tumors using RNA-sequencing (RNA-seq).

## Results

### SCAN-B cohort

SCAN-B (ClinicalTrials.gov identifier NCT02306096) is an ongoing, large, population-based breast cancer study started in 2010 and now enrolling patients at nine hospitals in Sweden, wherein all newly diagnosed patients are offered to participate^44,45^. From this cohort, we analyzed RNA-seq data from 3207 patients with longer follow-up (diagnosed between 1 September 2010 and 31 March 2015). The present cohort is a subset of the previously described cohort of 3217 patients^46–48^, which has been reduced to 3207 samples due to additional quality controls. The clinical characteristics are presented in Table 1 and are in concordance with the typical clinicopathological properties of breast cancer patients in Sweden. RNA-seq-based gene expression data was used to determine the PAM50 molecular subtypes of the tumors: 48% were classified as luminal A, 28% luminal B, 8.7% HER2-enriched, 9.9% basal-like, and 3.5% normal-like. Endocrine treatment was administered to 78.0% (n = 2502) of the patients in the cohort, out of which 218 patients also received chemotherapy.

**Table 1:** Clinicopathological parameters of the SCAN-B cohort.

	Sample Number (n = 3207) (%)	*ESR2*-High (n = 1069)	*ESR2*-Low (n = 2138)	*ESR2* High vs Low (p-value)
**Patient age (years)**				
Median (range, SD)	64 (24–96, 13.2)	63 (24–96, 13.2)	65 (24–95, 13.1)	0.00014
< 50 years old	328 (10.2%)	134 (12.5%)	194 (9.1%)	0.003
> 50 years old	2878 (89.8%)	934 (87.3%)	1944 (90.9%)	
Missing	1 (.03%)	1 (.09%)	0 (0%)	
**Tumor Size (mm)**				
Median (range, SD)	17 (1–126, 12.1)	17 (1–125, 11.5)	17 (1–126, 12.4)	0.016
**Lymph Node status**	**N0 vs N1-3**			0.0018
N0	2734 (85.3%)	881 (82.4%)	1853 (86.7%)	
N1 N3	457 (14.3%)	182 (17%)	275 (13%)	
Missing	16 (0.5%)	6 (0.6%)	10 (0.5%)	
**Ki67 Status**	**Ki67 Low vs High**			0.38
Low	267 (8.3%)	96 (9%)	171 (8%)	
High	883 (27.5%)	291 (27.2%)	592 (27.7%)	
Missing	2057 (64.1%)	682 (63.8%)	1375 (64.3%)	
**Nottingham Histological Grade**	**G1 vs G2 vs G3**			0.3
G1	481 (15%)	166 (15.5%)	315 (14.8%)	
G2	1504 (47%)	478 (44.7%)	1026 (48%)	
G3	1158 (36.1%)	397 (37.1%)	761 (35.6%)	
Missing	64 (2%)	28 (2.6%)	36 (1.7%)	
**PAM50 Subtypes**	**Luminal(A + B) vs Basal vs HER2-enriched**			2.905E−016
Luminal A	1540 (48%)	529 (49.5%)	1011 (47.3%)	
Luminal B	896 (28%)	165 (15.4%)	731 (34.2%)	
Basal-like	317 (9.9%)	159 (14.9%)	158 (7.4%)	
HER2-enriched	278 (8.7%)	117 (11%)	161 (7.5%)	
Normal-like	112 (3.5%)	75 (7%)	37 (1.7%)	
Missing	64 (2%)	24 (2.2%)	40 (2%)	
**ERα-status**	**ERα-positive vs ERα-negative**			2.163e−11
Positive	2715 (84.7%)	838 (78.4%)	1877 (87.8%)	
Negative	475 (14.8%)	223 (20.9%)	252 (11.8%)	
Missing	17 (0.5%)	8 (0.75%)	9 (0.42%)	
**Clinical Groups**	**ERnHER2p vs ERpHER2n vs ERpHER2p vs TNBC**			4.4e−10
ERα-negative HER2-positive	124 (3.9%)	62 (5.8%)	62 (3%)	
ERα-positive HER2-negative	2308 (72%)	696 (65.1%)	1612 (75.4%)	
ERα-positive HER2-positive	287 (9%)	100 (9.4%)	187 (8.7%)	
TNBC	320 (10%)	146 (13.7%)	174 (8.1%)	
Missing/Unclassified	168 (5.2%)	65 (0.06%)	103 (4.8%)	
**Histopathological type**	**Ductal vs Lobular**			0.002
Ductal	2596 (80.1%)	841 (78.7%)	1755 (82.1%)	
Lobular	383 (12%)	155 (14.5%)	228 (10.7%)	
Both ductal and lobular cancer	50 (1.6%)	22 (2%)	28 (1.3%)	
Cancer in situ only	0 (0%)	0 (0%)	0 (0%)	
Other invasive cancer	142 (4.4%)	33 (3.1%)	109 (5.1%)	
Both invasive and cancer in situ	3 (0.09%)	3 (0.3%) 0 (0%)		
Missing	33 (1%)	15 (1.4%)	18 (0.8%)	
**Therapy Received**	**Endocrine vs Chemo**			0.0013
Endocrine Therapy	2502 (78%)	773 (72.3%)	1729 (81%)	
Chemotherapy	1258 (39.2%)	455 (42.6%)	803 (37.5%)	

Clinicopathological information was retrieved from the Swedish National Quality Register for breast cancer via SCAN-B. Variables are defined as in the standard Swedish clinical routine, with Ki67 status determined using local cut-offs. PAM50 subtyping is derived from the RNA-sequencing data as described in Brueffer et al.^46,47^. Significant p-values are underlined.

### *ESR1* and *ESR2* mRNA expression in SCAN-B breast tumor tissues 

Quantification of *ESR2* mRNA levels in transcripts per million mapped reads (TPM) across the entire SCAN-B dataset revealed a generally low expression. The median expressi on wa s 0.05 log_2_(TPM + 0.1), and in 1027 samples *ESR2* was not expressed at all. We stratified the cohort into two groups based on *ESR2* expression levels (upper tertile: “*ESR2*-high”; lower two tertiles: “*ESR2*-low”) and performed statistical two-group comparisons for relevant clinical factors between the *ESR2*-high and *ESR2*-low groups. Twice as many samples of basal subtype and ERα-negative status could be found in the *ESR2*-high group as compared to the *ESR2* - low group. Tumor grade and Ki67 status did not differ between the *ESR2*-high vs -low subgroups. Median age and median tumor size differed between the *ESR2*-high vs -low subgroup s (p  < 0.05) (Table 1).

**Figure 1:**
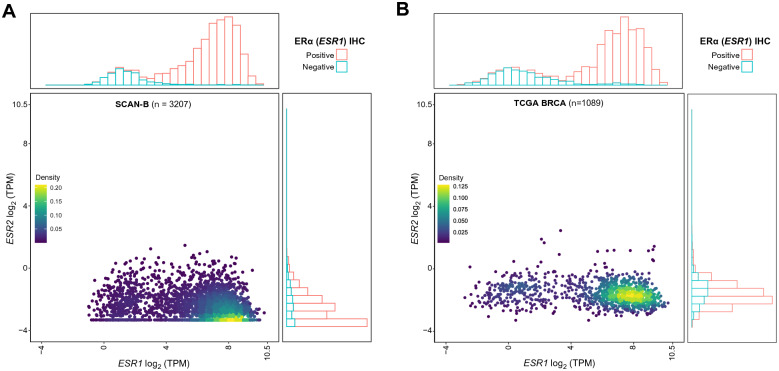
*ESR1* and *ESR2* mRNA expression in SCAN-B and TCGA data sets. (**A**) Scatterplot of *ESR1* and *ESR2* mRNA expression (log_2_TPM). *ESR1* and *ESR2* data points are colored by density using 2D kernel density estimation function from the MASS R package. Adjacent to the scatterplot, histograms are shown indicating the frequency of expression values and color-coded according to ERα clinical status. (**B**) Expression of *ESR1* and *ESR2* is shown for the TCGA dataset as in panel A.

We investigated the expression patterns of *ESR1* and *ESR2* according to clinical ERα status (positive or negative). *ESR1* followed the well-known bimodal distribution pattern^49,50^, whereas the expression of *ESR2* was very low and exhibited a left-skewed distribution (Fig. 1A). The two genes showed a weak inverse correlation (Spearman rank correlation test R = −0.18, p = 2e−10), consistent with a prior report^49^. Of the 475 ERα-negative tumors, 46.9% (n = 223) were classified as *ESR2*-high, and among the 2715 ERα-positive cases, 30.9% (n = 838) were classified *ESR2*-high.

**Figure 2:**
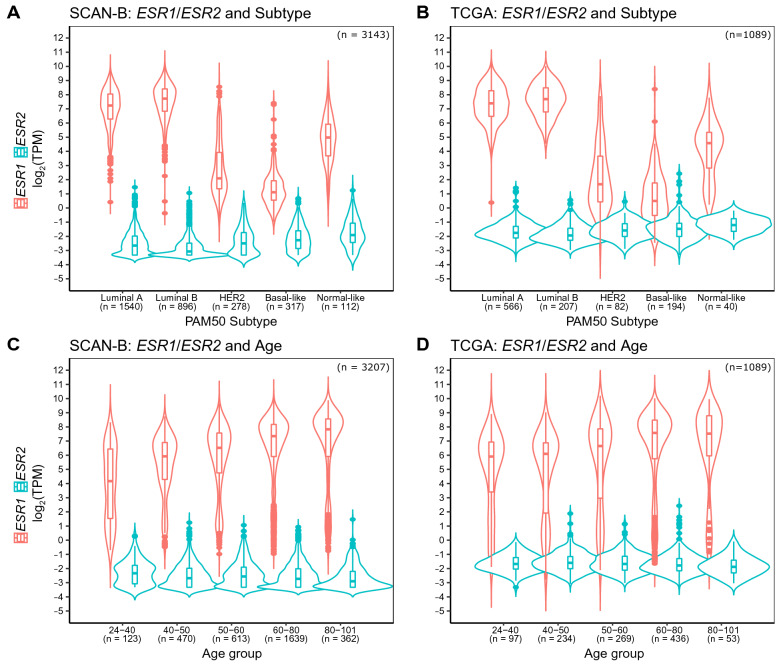
*ESR2* mRNA by PAM50 molecular subtype in SCAN-B (**A**) and TCGA (**B**) cohorts, and by age groups (age at diagnosis) in SCAN-B (**C**) and TCGA (**D**).

We compared the relative expression of the estrogen receptor genes across molecular subtypes. Within the SCAN-B data set, median *ESR2* expression followed the trend: normal-like > basal-like > HER2-enriched > luminal A > luminal B, being highest in normal-like tumors (Fig. 2A). Conversely, the median *ESR1* expression was highest in luminal B and lowest in the basal-like subtype (luminal B > luminal A > normal-like > HER2-enriched > basal-like). We also analyzed the expression levels across patients stratified by age at diagnosis (Fig. 2C). Median *ESR1* expression increased with increasing patient age at diagnosis (Spearman correlation R =  + 0.28, p = 2.2e−16), whereas median *ESR2* mRNA quantities remained largely stable across age groups (Spearman correlation R = −0.078, p = 1.1e−05).

### High *ESR2 *expression is associated with better prognosis for patients receiving endocrine therapy and in triple-negative disease

**Figure 3:**
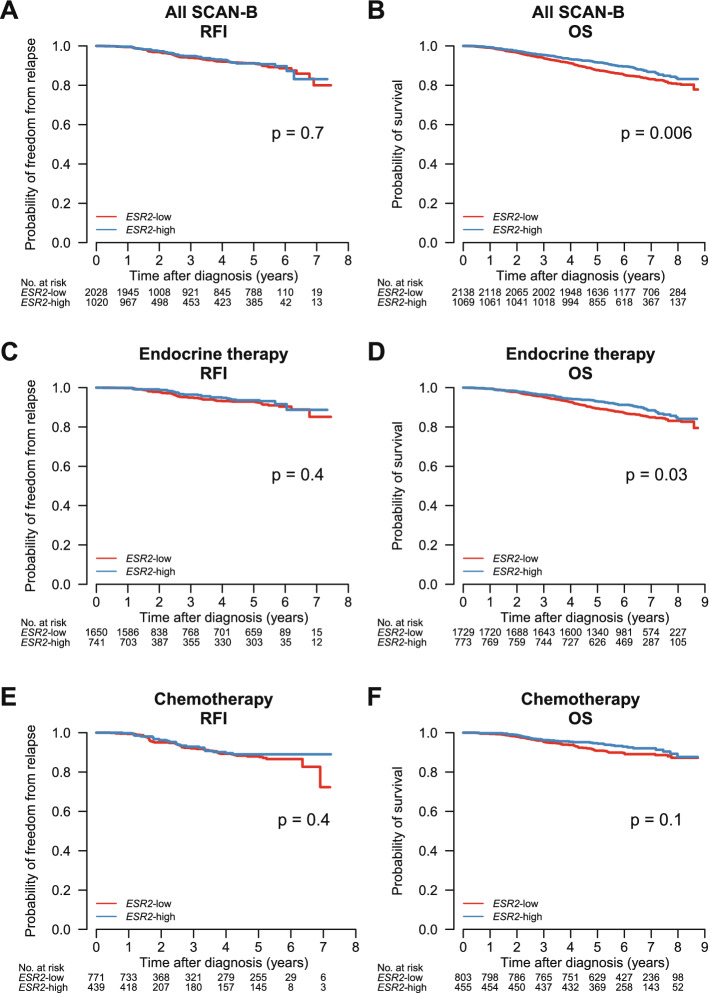
*ESR2* expression and association to overall survival (OS) and relapse-free interval (RFI) in the full SCAN-B cohort (**A**,**B**), the endocrine-treated sub-group (**C**,**D**) and the chemotherapy-treated subgroup (**E**,**F**).

We analyzed patient outcome regarding overall survival (OS) and relapse-free interval (RFI). The median follow-up time was 6.2 years. We found that high *ESR2* expression was not associated with improved RFI (Fig. 3A), however was associated with improved OS (logrank test p = 0.006; Fig. 3B). These results are in concordance with another study where higher levels of ERβ were found to be associated with favorable OS in inflammatory breast cancer patients^51^.

**Figure 4:**
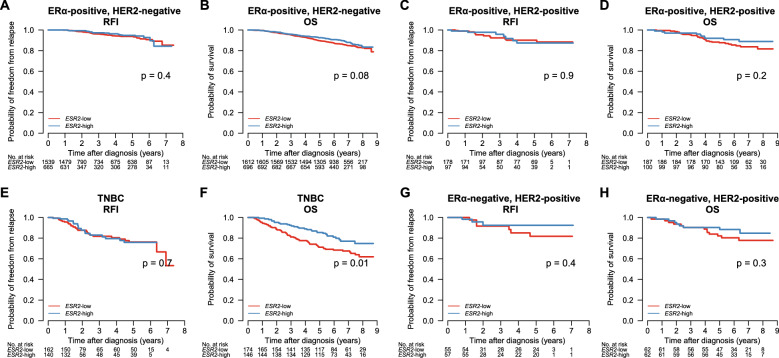
*ESR2* expression and association to overall survival (OS) and relapse-free interval (RFI) in the SCAN-B clinical groups. (**A,B**) Patients with ERα-positive, HER2-negative breast cancer; (**C,D**) ERα-positive, HER2-positive breast cancer; (**E,F**) Triple-negative breast cancer (TNBC); and (**G,H**) ERα-negative, HER2-positive breast cancer.

We also analyzed outcome for the sub-group of patients that received endocrine therapy (ET) with or without other systemic therapies (n = 2502) and for the patients receiving chemotherapy with or without other systemic therapy (n = 1258). *ESR2* expression was not associated with RFI outcome (Fig. 3C), but higher *ESR2* expression was associated with better OS in the endocrine-treated group (logrank test p = 0.03; Fig. 3D). No significant associations to RFI and OS were found in the patients who received chemotherapy (Fig. 3E, 3F). Furthermore, patients were stratified based on clinical groups: ERα-positive/HER2-negative (n = 2308), ERα-positive/HER2-positive (n = 287), ERα-negative/HER2-positive (n = 124) and triple-negative breast cancer (TNBC, n = 320). We found that low *ESR2* expression was associated with poor OS in TNBC (logrank p = 0.01), but not in the other clinical sub-groups (Fig. 4).

**Figure 5:**
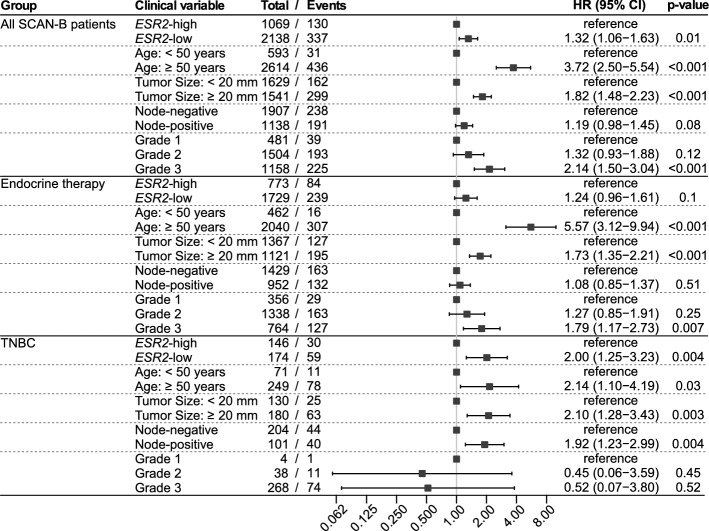
Multivariate analysis of high *ESR2* expression in the full SCAN-B cohort, the endocrine-treated group, and the triple-negative breast cancer (TNBC) group.

To further examine our findings that high *ESR2* expression is associated with improved OS, we performed Cox regression multivariable analysis, adjusting for age, tumor size, lymph node status, and grade (Fig. 5). In the full cohort, *ESR2* expression remained a significant prognostic factor with hazard ratio (HR) 1.34 (95% CI 1.06–1.32; p = 0.01). For patients receiving endocrine therapy, low *ESR2* expression carried an HR of 1.24 (95% CI 0.96–1.61; p = 0.1, not significant). In triple-negative tumors, low expression of *ESR2* exhibited an increased HR of 2.0 (95% CI 1.25–3.23; p = 0.004).

### Validation of findings in the TCGA dataset

We set out to validate our results in the TCGA breast cancer cohort^52^. Albeit not population-based, and as previously reported, having a bias towards larger tumors with higher grade and stage^53^, this dataset represents a comparably large tumor collection with publicly available RNA-seq data. Generally, the SCAN-B results were confirmed in TCGA. *ESR2* mRNA overall showed low expression across TCGA. As expected, in TCGA *ESR1* followed a bimodal distribution pattern in the histogram (Fig. 1B). Moreover, *ESR1* and *ESR2* levels showed a weak inverse correlation (Fig. 1B, Spearman correlation R = −0.20, p = 3.5e−12). The *ESR2*-high group was comprised of 29% of the ERα-positive tumors (231/800), as compared to 48% ERα-negative tumors (115/239). Expression of *ESR2* in TCGA followed the same trend as in SCAN-B with higher expression in normal-like, basal-like, and HER2-enriched groups and lower expression in the luminal subtypes (Fig. 2B). While there were small but significant differences in *ESR2* expression across molecular subtypes in SCAN-B we did not find significant differences in TCGA (Supplementary Table S1). *ESR1* and *ESR2* expression patterns in the TCGA cohort followed same trend as in SCAN-B, where *ESR1* expression increased with patient age at diagnosis but *ESR2* expression was largely stable across age groups (Fig. 2D).

OS and RFI for the TCGA breast tumors were analyzed using patient survival at 10 years, after which all events were censored, for comparison with the SCAN-B cohort. The median follow-up time was 2.3 years. As with the SCAN-B dataset, patients were subdivided based on treatment received; endocrine therapy with or without other systemic treatment (n = 524) and chemotherapy with or without other treatment (n = 576). Within these groups we could not find any association with outcome for OS, as we had seen in the SCAN-B cohort (Supplementary Figure S1). However, when analyzing outcome in the clinical subgroups, we found that, in contrast to our findings in the SCAN-B cohort, OS and RFI were significantly improved for *ESR2*-high patients in the ERα-negative HER2-positive subgroup (RFI, p = 0.02, OS, p = 0.03; Supplementary Figure S2).

### Differential gene expression and GSEA analysis of *ESR2 *high vs low groups

To shed light on the potential biology behind the differences in outcome of *ESR2* expression groups, we next performed differential gene expression (DGE) analysis for tumors with *ESR2*-high versus *ESR2*-low expression to determine the genes co-modulated with *ESR2* within the SCAN-B cohort. To remove the influence of ERα effects, which are known to have a strong impact on global gene expression patterns, we performed separate DGE analyses within the ERα-positive and -negative sub-groups. We applied a false discovery rate (FDR) cut-off of ≤ 0.05 and identified up- and down-regulated genes according to the log_2_ fold change (log_2_FC) with the criteria log_2_FC ≥  1 .5 for up-regulated genes and log_2_FC ≤ − 1.5 for down-regulated genes. Within the ERα-positive subgroup, a total of 64 genes were found to be upregulated in *ESR2*-high vs -low tumors, and 6 genes were downregulated. In the ERα-negative subgroup, 199 genes were upregulated and 22 genes were downregulated in *ESR2*-high vs -low cases.

 Of all the genes identified in the DGE analyses, a total of 42 up-regulated genes (*BANK1*, *BLK*, *CCL19*, *CD19*, *CD79*, *IGLL5*, *IRF4*, *JCHAIN*, *PAX5*, *TCL1A*, *TNFRSF17*, *VPREB3* and others) were found to be upregulated in *ESR2*-high tumors in both the ERα-positive and -negative subgroups, and two down-regulated genes (*COL11A1*, *EEF1A2*) were found to be common between these subgroups (Supplementary Table S2; common genes highlighted). Up-regulated genes were found to be involved in processes such as immune response, B-cells signature (*CCL19, JCHAIN, VPREB3, IGLL5, CD19, BLK, IGHD, CD79A*), chromosomal rearrangement (*TCL1A, TNFRSF17, IRF4, PAX5*) as well as proto-oncogenes such as *TCL1A, PAX5*, which have been shown to be potent regulators of malignant processes in breast cancer^54–57^.

**Figure 6:**
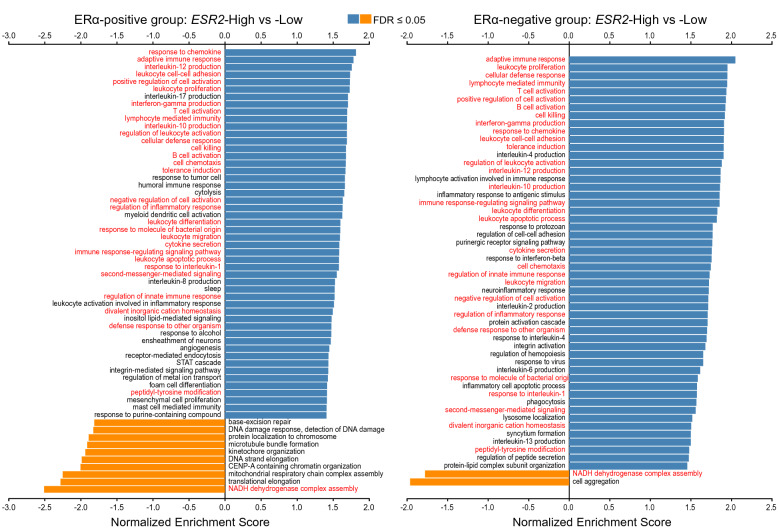
Gene Set Enrichment Analysis (GSEA; GO category: Biological Process) of genes ranked by fold change (log_2_FC) and p-value < 0.05, associated with *ESR2*-high vs -low in SCAN-B. GSEA analysis based on *ESR2*-high vs -low was performed separately for the ERα-positive (**A**) and ERα-negative (**B**) subgroups. Categories found enriched in both subgroup analyses are indicated by red text.

Next, we performed gene set enrichment analysis (GSEA) to find the statistically significant, concordant gene sets that differed between *ESR2*-high vs -low in both ERα-positive and -negative tumors. The log_2_FC ranked gene expression values were analyzed for enrichment within Gene Ontology (GO) category ‘non-redundant biological processes’. Most of the GO categories enriched within ERα-positive and ERα-negative subgroup analyses were found to be shared, with a common theme related to immune system modulation including the positively enriched GO categories immune responses, B cell activation and proliferation, response to chemokine, and cellular defense (Fig. 6). The genes involved in these positively enriched GO categories within the ERα-positive and -negative subgroups were also upregulated in the ERα-positive and -negative DGE list (Supplementary Tables S3 and S4). Genes involved in negatively-enriched GO categories such as NADH dehydrogenase complex assembly were also found to be common within the ERα-positive and -negative subgroups. Other GO categories negatively enriched in *ESR2*-high were unique within the ERα-positive subgroup analysis (such as base-excision repair, DNA damage response, protein localization to chromosome, microtubule bundle formation, kinetochore organization, and DNA strand elongation) or within the ERα-negative subgroup analysis (cell aggregation) (Fig. 6).

Importantly, the common positively enriched categories between the two ERα groups were associated with a myriad of immune response processes such as adaptive immune response, cellular defense response, lymphocyte mediated immunity, leukocyte cell–cell adhesion and proliferation, B and T cell activation, production of interleukins, cell killing, cellular defense response, and regulation of inflammatory response. Together, this raises the hypothesis that the improved survival for *ESR2*-high tumors may be partly associated with the local and systemic immune response.

## Discussion

In this study we have characterized the expression of *ESR2* mRNA using RNA-seq analysis of a large cohort of breast cancer samples from SCAN-B. Our analyses revealed that *ESR2* transcripts are generally much less abundant than *ESR1* across all breast cancers. Within this general low expression, *ESR2* expression was highest in the ERα-negative subtypes (normal-like, basal-like, and HER2-enriched) and lower in the ERα-positive subtypes (luminal A and luminal B). The relatively higher expression in basal-like subtype may be of clinical interest, since some studies report that ERβ expression in ERα-negative tumors may be a predictor for response to endocrine therapy in these patients^30^; our results support this conclusion.

We also found that higher expression of *ESR2* was associated with better OS for patients treated with endocrine therapy, although the effect did not remain significant when adjusted for other clinical variables. Interestingly, the clinical subgroup analyses revealed that the overall survival effect was most pronounced in the TNBC subgroup. In the SCAN-B cohort, we could not observe any association of *ESR2* expression with RFI, which may be an effect of shorter follow-up times compared to OS.

Analysis of the TCGA breast tumors confirmed that *ESR2* was generally expressed at low levels, but higher in ERα-negative PAM50 subtypes. For association of *ESR2* expression and improved OS, the results within the entire SCAN-B cohort and TCGA cohort showed a similar trend, with SCAN-B showing a significant association whereas in TCGA, the survival curves had a later separation that did not reach statistical significance. The SCAN-B results on patient OS following endocrine treatment or in TNBC were not reproduced. There could be several reasons for these discrepancies. First, due to smaller sample size in TCGA, a potential survival association may not be as readily detectable. Furthermore, TCGA is not a population-based cohort, but rather spans samples collected from large number of clinical sites, varying timeframes of diagnosis, various treatment regimens of diverse countries, and furthermore is biased towards more advanced tumors^52^. It may be that this heterogeneity affects the analysis for patient outcome within TCGA. To note, we did find that *ESR2*-high patients had a significantly improved OS and RFI in TCGA HER2-positive patients, which we did not observe in the SCAN-B cohort. Eighty percent of HER2-positive patients in SCAN-B received anti-HER2 therapy, compared to 28% in TCGA (note, 37% of HER2-positive patients were missing treatment information) and this may have improved the overall outcome for the SCAN-B group. A potential weakness of our study is that it relies on the quantities of mRNA rather than protein. The global concordance of mRNA to protein is expected to be high, with a commonly stated correlation of 0.6^58^, but it does not completely explain the variance in protein levels, which are also affected by translation, post-translational modifications, and regulation of the rate of protein decay. Consequently, our study must be interpreted in the context of the biological phenotype related to high *ESR2* mRNA expression. On the other hand, our approach allows us to circumvent the problematic use of ERβ antibodies, which have been shown to be exceedingly unreliable to date^42,43^.

Another possible limitation of our study is its reliance on RNA-seq of bulk tumor tissue samples. Since bulk RNA-seq mainly reflects the averaged gene expression across thousands of cells at different transcriptomic states or even different cell types within the same tissues (for example, infiltrating immune cells or normal cells in tumor samples), it is not possible to determine from which compartment in the tumor or tumor microenvironment the gene expression signals originate. It is possible that, along with BC cells, immune cells such as lymphocytes may be contributing to *ESR2* expression, which has also been shown in previous studies^59–61^. Indeed, our DGE analyses demonstrate the enrichment of lymphocytic markers in the upregulated gene lists within ERα-positive and -negative subgroups. This may suggest that *ESR2* is co-expressed within the immune cell compartment, or that *ESR2* is expressed in the tumor compartment and is associated to signatures of immune cell infiltration. Additional studies at the protein level, or using approaches such as single-cell sequencing, will be needed to further decipher the origin of the *ESR2* expression signature.

GSEA analysis revealed co-expressed genes, many of which were enriched in immune response biological processes and pathways in both ERα-positive and ERα-negative tumors. This robust result may suggest that the improved survival seen in patients with *ESR2*-high expression could be related to the local and systemic immune response. In this respect, *ESR2* may be an active participant or be an associated biomarker for immune cell activity.

TNBC accounts for approximately 10–15% of all breast cancers^62^, which lacks expression of ERα, PR, HER2^63,64^. TNBCs are associated with aggressive features, do not benefit from treatments with targeted therapies currently used, and have poorer prognosis^65^. Our analysis showed that *ESR2*-high tumors had favorable OS (p = 0.006), *ESR2* expression was high in the basal-like tumors, associated with better OS in TNBC (p = 0.01), and associated to immune response in GSEA analysis. Taken together, these results indicate that ERβ could be an interesting biomarker for more favorable-prognosis TNBC, a target for re-activation, possibly providing alternative therapeutic options for patients with TNBC.

In conclusion, we have characterized the expression of *ESR2* across the largest population-based breast cancer cohort to date, and described its association to clinicopathological parameters and patient outcomes. We found that *ESR2* mRNA is not abundantly expressed in primary breast cancer, but that higher *ESR2* expression is found particularly within ERα-negative breast cancer subtypes and that *ESR2*-high has a significant association to survival in endocrine-treated patients as well as patients with TNBC. Our study brings further clarity to the ERβ/*ESR2* field of research and sets the stage for further exploration of this poorly understood receptor.

## Materials and methods

### Patient enrollment and study design

The study was conducted in accordance with the Declaration of Helsinki and was approved by the Regional Ethics Review Board of Lund at Lund University (diary numbers 2007/155, 2009/658, 2009/659, 2010/383, 2012/58, 2013/459), the county governmental biobank center, and the Swedish Data Inspection group (diary number 364–2010). Trained health professionals provided the written information and all patients gave written informed consent.

Clinical/medical records were retrieved from the Swedish National Cancer Registry (NKBC). The median overall follow-up time for the early BC patients in the SC AN-B cohort was 6.2 years (IQR = 2.2). Hormone receptor positive early breast tumors were defined as cases expressing estrogen (ERα) or progesterone (PR) receptors using an immunohistochemical staining cutoff ≥ 10% of neoplastic/BC cells as indicated by Swedish guidelines and HER2 status was assessed according to standard recommendations^66^.

### Tumor processing and RNA-seq gene expression measurements

SCAN-B tissue collection, tumor sample processing, preservation in RNA-later, mRNA enrichment by poly-A selection, mRNA-sequencing and read processing were performed as described previously^44,46,47^.

In brief, the RNA-seq data was processed through an automated multistep analysis pipeline implemented in BASE^67,68^ with extension package Reggie^69^. Picard toolkit^70^ v2.22.3 was used for demultiplexing raw sequencing read data using tools *ExtractIlluminaBarcodes* and *IlluminaBasecallsToFastq* with default parameters except –*INCLUDE_NON_PF_READS* = *false*. Trimmomatic^71^ v0.33 with the recommended parameters for PE reads was used to remove adaptor sequences and poor-quality reads (ILLUMINACLIP:TruSeq3-PE-2.fa:2:30:12:1:true; MINLEN:20; MAXINFO:40:0.9 and MINLEN:20). Each data set was filtered to remove reads that align (using Bowtie2^72^ v2.2.9 with default parameters except *-k 1 –phred33 –local*) to ribosomal RNA/DNA (GenBank loci NR_023363.1, NR_003285.2, NR_003286.2, NR_003287.2, X12811.1, U13369.1), phiX174 Illumina control (NC_001422.1), and sequences contained in the UCSC hg38 RepeatMasker track.

Reads were aligned using HISAT2^73^ v2.1.0 to the human genome reference GRCh38/hg38 using the GENCODE release 27 transcriptome model, with default parameters except –*no-unal* –*non-deterministic* –*novel-splicesite-outfile* ${SPLICEFILE} –*rna-strandness RF*. HISAT2 indexes were created using the –*snp* parameter and dbSNP build 150. StringTie^74^ v1.3.3b was used to calculate expression levels as fragments per kilobase of transcript per million mapped reads (FPKM), with default parameters including –*rf* -*e* using protein coding transcripts from GENCODE release 27 as transcriptome model. Novel transcripts were discarded. An FPKM gene expression matrix was generated from .ctab files using tximport^75^ and subsequently transformed to TPM values. TPM values were log_2_ transformed. To avoid zero values and large negative values in log_2_ transformation, a fixed pseudo-count of 0.1 was added to all transcripts in the TPM matrix prior to transformation. Molecular subtyping using the PAM50 gene list was performed as described previously^44^. All data are available from the NCBI Gene Expression Omnibus (Accession No. GSE96058).

### Validation using TCGA-BRCA cohort

TCGA clinical and expression data was obtained from the GDC Legacy Archive (https://portal.gdc.cancer.gov) and accessed using TCGABiolinks^76^. The TCGA BRCA samples were filtered for distinct barcodes (n = 1222), only primary tumor samples (n = 1102), and female gender (n = 1089). Gene expression data was obtained as FPKM, converted to TPM, and transformed using log_2_(TPM + 0.1) for use in gene expression analysis.

### Statistical analysis

All analyses were performed using R 3.6.1. P values of ≤ 0.05 were considered significant. Spearman rank correlation was used to determine correlations between expression of *ESR1* and *ESR2*. Since the data was not normally distributed, Kruskal–Wallis non-parametric test (for significant difference between groups) as well as Wilcoxon rank sum test (for multiple pairwise comparisons between groups) were used to compare and plot expression of the *ESR1* and *ESR2* genes in various clinical groups such as PAM50 subtype and age groups in both the SCAN-B and TCGA cohorts. To evaluate significant differences in the clinicopathological variables for the *ESR2*-high and *ESR2*-low groups, Mann Whitney U test (for continuous variables) and Fisher’s exact test (for categorical data) were used. DGE was performed using the limma-voom package^77^ in R. GSEA was performed using the fgsea package^78,79^ in R as well as WebGestalt^80–82^.

Patients were sub-grouped according to the treatment received (endocrine therapy with or without other treatments and chemotherapy with or without other treatments) for both SCAN-B and TCGA cohorts. Patients were also subdivided based on receptor status for ER, PR and HER2, resulting in four clinical groups: (1) ERα-positive, HER2-negative (PR-positive or -negative), (2) ERα-positive, HER2-positive (PR-positive or -negative), (3) ERα-negative, HER2-positive (PR-positive or -negative) and (4) triple-negative (TNBC): ERα-negative, PR-negative, and HER2-negative.

### Survival analysis

For SCAN-B, overall survival (OS) outcome was defined as death from any cause and the relapse-free interval (RFI) endpoint as locoregional or distant recurrence. For TCGA, OS and RFI were calculated as described earlier^83^ with a modification to RFI calculation. For patients having new tumor event, only local recurrence and distant metastasis were taken into account as endpoints. Survival analysis was performed by Kaplan–Meier and Cox regression survival analyses. Transformed *ESR2* expression data was divided into tertiles, with the first tertile defined as *ESR2*-high, and the bottom two tertiles at *ESR2*-low.

Proportional hazards assumptions were checked graphically by Schoenfeld residual plots. One of the variables in the multivariable model for the full SCAN-B cohort, Nottingham Histological Grade (NHG), showed a time varying effect. Therefore, three models were fitted to estimate the adjusted effect of *ESR2* on outcome, a model with adjustment for NHG despite its non-proportional effect on outcome, a model with stratification for NHG, and finally a model with interaction between NHG and follow-up time allowing for a time dependent effect of NHG on outcome. The estimated HRs for *ESR2* status were essentially the same in these three models (range 1.30–1.32).

## Supplementary Information

Supplementary Information 1.

Supplementary Information 2.

Supplementary Information 3.
