# Supplementary material for: Clinical associations of ESR2 (estrogen receptor beta) expression across thousands of primary breast tumors
Source: Sci Rep. 2022 Mar 18;12:4696. doi: 10.1038/s41598-022-08210-3 (PMC8933558; doi:10.1038/s41598-022-08210-3)
Supplement: Supplementary file 1 — Supplementary Information 1. [file 41598_2022_8210_MOESM1_ESM.pdf]

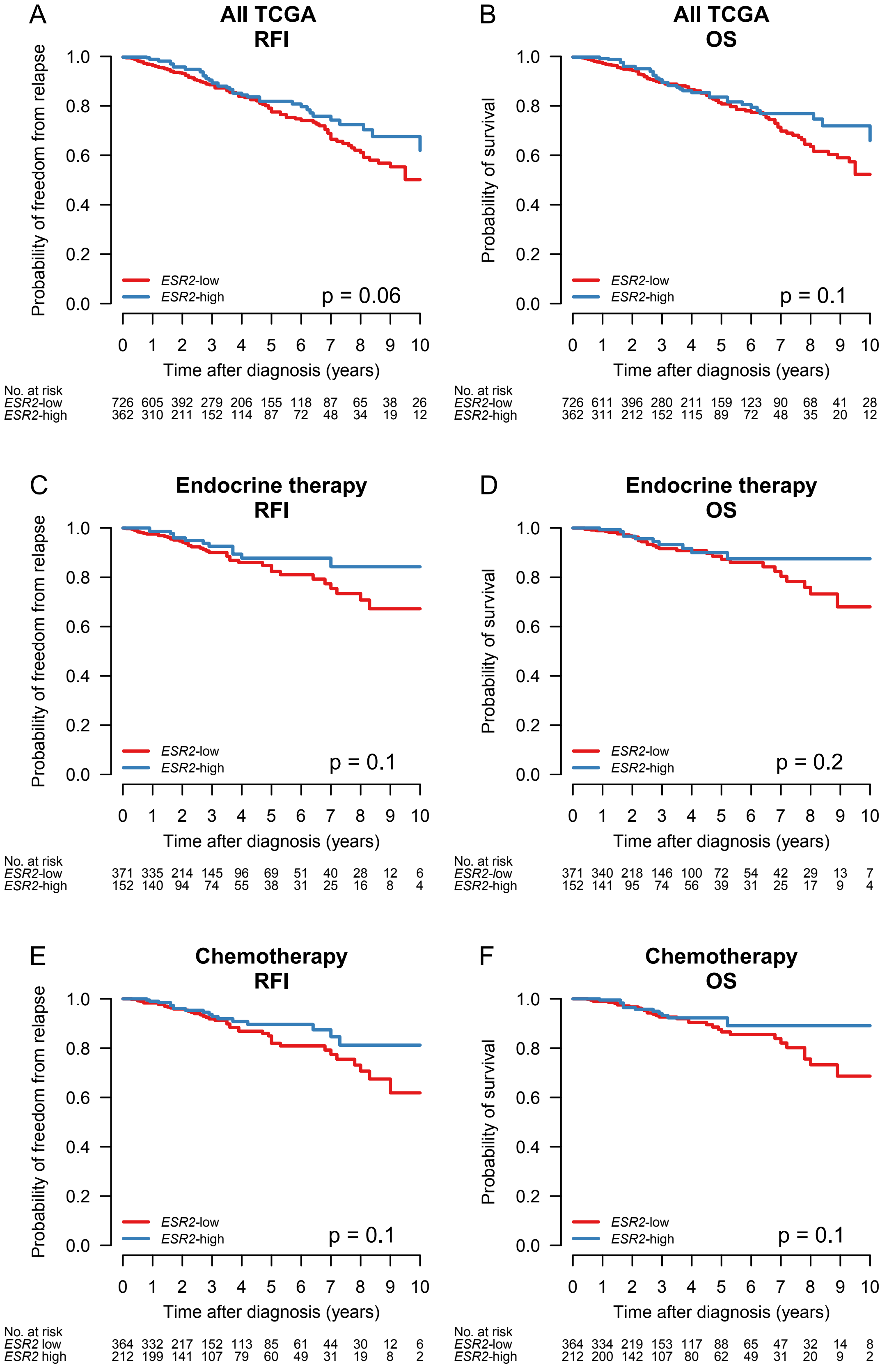

Supplementary Figure S1: *ESR2* expression and association to overall survival (OS) and relapse-free interval (RFI) in the full TCGA cohort (**A, B**), the endocrine-treated sub-group (**C, D**) and the chemotherapy-treated subgroup (**E, F**).
