# Supplementary material for: Clinical associations of ESR2 (estrogen receptor beta) expression across thousands of primary breast tumors
Source: Sci Rep. 2022 Mar 18;12:4696. doi: 10.1038/s41598-022-08210-3 (PMC8933558; doi:10.1038/s41598-022-08210-3)
Supplement: Supplementary file 2 — Supplementary Information 2. [file 41598_2022_8210_MOESM2_ESM.pdf]

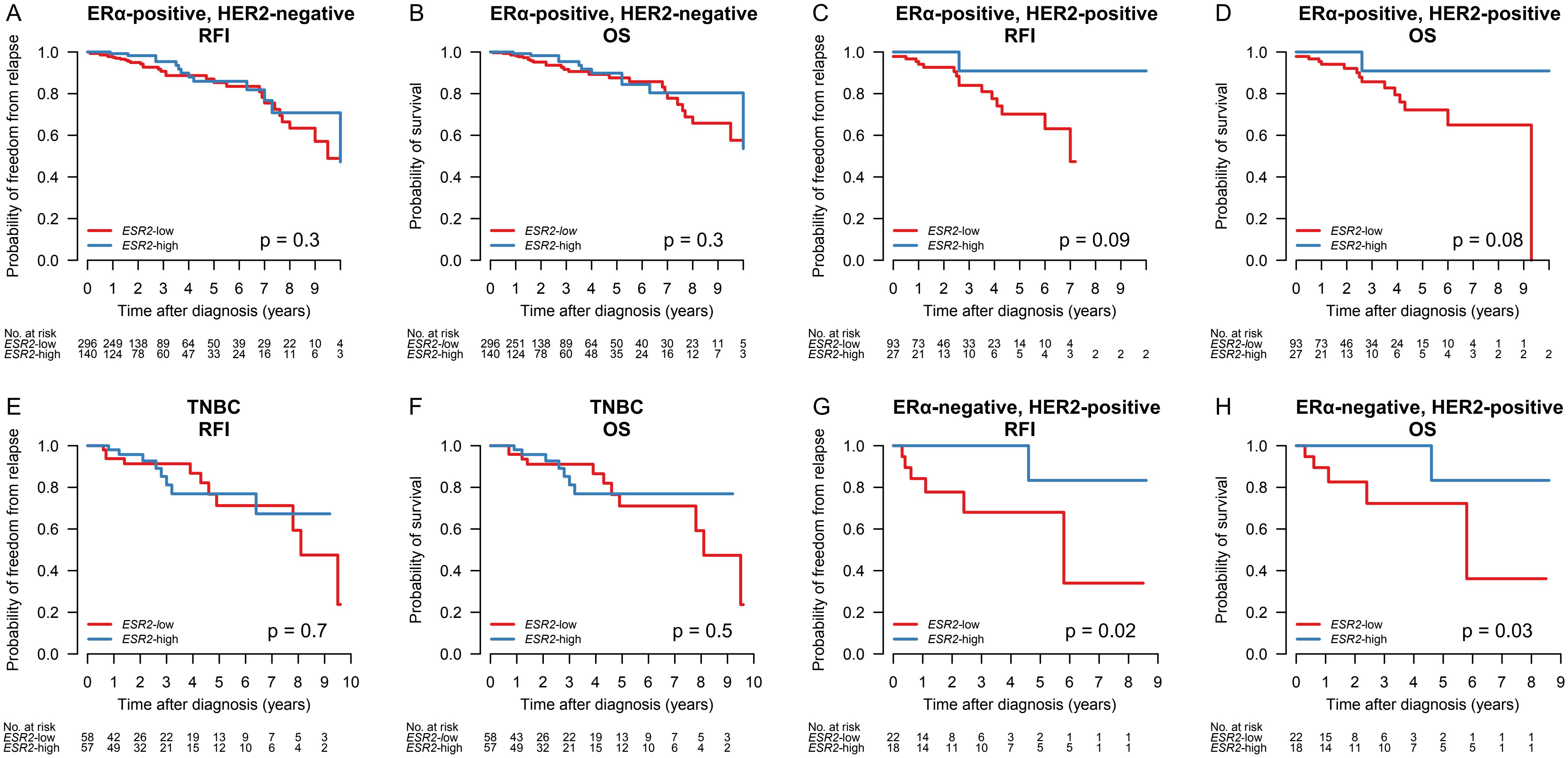

Supplementary Figure S2: *ESR2* expression and association to overall survival (OS) and relapse-free interval (RFI) in the TCGA clinical groups. **A-B**) Patients with ERα-positive, HER2-negative breast cancer; **C-D**) ERα-positive, HER2-positive breast cancer; **E-F**) Triple-negative breast cancer (TNBC); and **G-H**) ERα-negative, HER2-positive breast cancer.
